# Sodium orthovanadate inhibits growth of acute leukemia HL60 cells and HL60/A cells *in vitro*

**DOI:** 10.1042/BSR20201918

**Published:** 2020-09-28

**Authors:** Lulu Zhang, Nan Wei, Guoying Guan, Tao Song, Yingying Xu, Shuye Wang, Jin Zhou

**Affiliations:** 1 Department of Gerontology, The First Affiliated Hospital of Harbin Medical University, Harbin 150001, China; 2 Department of Hematology, The First Affiliated Hospital of Harbin Medical University, Harbin 150001, China

**Keywords:** Acute promyelocytic leukemia, Apoptosis, Autophagy, Sodium orthovanadate

## Abstract

Vanadium is an ultratrace element. The transition metal vanadium, widely exists in the environment and exhibits various biological and physiological effects in the human body, yet with no presently known specific physiological function in mammals. Sodium orthovanadate (SOV) is a kind of vanadium compound. SOV has shown promising antineoplastic activity in several human cancers. But the effects of SOV on acute promyelocytic leukemia (APL) are still unknown. In the present study, for the first time, we found that SOV could inhibit proliferation, induce G_2_/M cell cycle arrest and apoptosis, and could inhibit autophagy of acute leukemia cell lines *in vitro*. Thus, our findings suggest that SOV could effectively suppress the growth of acute leukemia HL60 cells and HL60/A cells through the regulations of proliferation, cell cycle, apoptosis and autophagy, and thus may act as a potential therapeutic agent in APL treatment.

## Introduction

Leukemia, a group of hematological malignancies, is one of the most dangerous threats to human health [1]. Acute myeloid leukemia (AML) is a group of heterogeneous diseases with the abnormally active proliferation of hematopoietic precursors, which block patients’ normal hematopoiesis, causing neutropenia and anemia [2,3]. Although more than 50% of adult AML patients show complete remission with conventional chemotherapeutic drugs, only 20–30% of patients show long-term disease-free survival [4]. Acute promyelocytic leukemia (APL), comprising 5–8% of cases of AML, is one of the best studied and understood hematopoietic malignancies [5–7]. Clinically, all-trans retinoic acid (ATRA) and arsenic trioxide (ATO) have been useful for curing the great majority of patients with APL. However, 10−30% of APL patients are not sensitive to ATRA and ATO [8]. Therefore, more effective therapeutic strategies for the treatment of APL are urgently needed.

Recently, vanadium has become more and more critical for the development and growth of some organisms as one of the dietary microelements. Vanadium salts have shown numerous biological activities, including anti-tumor activity against lung, kidney and prostate cancers [9,10]. They also exhibit antineoplastic activity against multidrug-resistant tumor cells, but the role of vanadium in leukemia has not yet been reported.

Autophagy is an evolutionarily conserved process involving lysosomal degradation of cytoplasmic and cellular organelles, which occurs in all eukaryotic cells from yeast to mammals. This process is believed to be important in the progression of cancers [11–17]. Our results show sodium orthovanadate (SOV) demonstrated dose-dependent inhibition of growth of the acute leukemia HL60 cells and HL60/A cells, and to the best of our knowledge, we initially found that SOV can inhibit autophagy and induce cell apoptosis. We concluded that SOV affects the growth of leukemia cells by inducing apoptosis rather than autophagy.

## Materials and methods

### Materials

SOV, rapamycin, and 3-methyladenine (3MA) were purchased from Sigma–Aldrich. The antibodies against BECN1, microtubule-associated protein 1A/1B-light chain 3 (LC3), cyclin B1, cdc2, poly (ADP-ribose) polymerase (PARP), caspase-3 were purchased from Cell Signaling Technology (Danvers, U.S.A.). The antibodies against β-actin, glyceraldehyde-3-phosphate dehydrogenase (GAPDH) were purchased from Santa Cruz Biotechnology (Santa Cruz, U.S.A.).

### Cell culture

The human HL60 and HL60/A cell lines were obtained from the American Type Culture Collection (Rockville, U.S.A.). HL60 and HL60/A cells were cultured in RPMI-1640 medium with 10% fetal bovine serum (FBS) (Gibco BRL, Rockville, MD, U.S.A.), 100 U/ml penicillin G and 100 μg/ml streptomycin at 37°C in a humidified 5% CO_2_ atmosphere. The logarithmic growth phase cells were used for further experiments.

### Cell viability assay

Cell viability was assessed by Cell Counting Kit-8 (CCK-8) kit (Dojindo Laboratories, Kumamoto, Japan). Briefly, the target cells were seeded on 96-well plates at a concentration of 3 × 10^3^ cells/well in RPMI-1640 medium and cultured overnight. The cells were then treated with increasing doses of vanadate for 72 h, and then the cell viability was assayed following the manufacturer’s protocol. The experiments were repeated three times.

### Cell cycle analysis

Cell cycle analysis was performed with a cell cycle kit (BD Biosciences, San Jose, California, U.S.A.) to determine the percentage of cells in the G_0_-G_1_, S and G_2_-M phases of the cell cycle. Briefly, the cells were harvested 48 h after treatment, and the number of cells was calculated. A total of 1 × 10^6^ cells were incubated with Reagents A–C according to the manufacturer’s instructions and subjected to flow cytometry. The experiments were repeated three times.

### Apoptosis analysis

We used two methods to detect apoptosis. The apoptotic rates of leukemia cell line cells after treatment were assessed using a PI/Annexin V-FITC apoptosis detection kit (BD Biosciences, San Jose, California, U.S.A.) according to the manufacturer’s instructions. Terminal deoxynucleotidyl transferase dUTP nick-end labeling (TUNEL) staining was performed using an *in situ* apoptosis detection kit (Roche, Shanghai, China) as described previously [18]. The experiments were repeated three times.

### Western blotting

SDS/PAGE and Western blots were performed as previously described [19]. In brief, cells or tumor tissues were homogenized in protein lysate buffer, and debris was removed by centrifugation. Samples containing 50 μg of total protein were resolved on 12% polyacrylamide SDS gels and electrophoretically transferred to polyvinylidene difluoride (PVDF) membranes. The membranes were blocked with 3% BSA, incubated with primary antibodies, and subsequently with an alkaline phosphatase-conjugated secondary antibody. They were developed with 5-bromo-4-chloro-3-indolyl phosphate/nitro blue tetrazolium (Tiangen Biotech Co., Ltd., Beijing, China). Anti-β-actin Ab and anti-GAPDH serve as an internal control for blot stain.

### Measurement of mitochondrial membrane potential

The lipophilic, cationic dye, JC-1, was used to measure changes in mitochondrial membrane potential (ΔΨm), as described previously [20]. Cells were incubated with 10 μg/ml of JC-1 for 20 min at 37°C in a 5% CO_2_ incubator, washed and resuspended in PBS at 1 × 10^6^ cells/ml, and then analyzed by flow cytometry as described previously [21] at an excitation wavelength of 514 nm. Data were collected at the emission wavelength of 529 nm (green fluorescence) of the JC-1 monomer and 585 nm (red fluorescence) for JC-1 aggregates. The ratio of red/green fluorescence intensities was recorded, and the relative ΔΨm was calculated according to the formula: experimental ratio value/control ratio value × 100.

### Statistical analysis

All the data are expressed as mean values ± standard deviation (SD). Analysis of variance (ANOVA) and Student’s *t* test was used to evaluate statistical significance. A value of less than 0.05 (*P*<0.05) was used for statistical significance.

## Results

### Inhibitory effect of SOV on proliferation in HL60 cells and HL60/A cells

**Figure 1:**
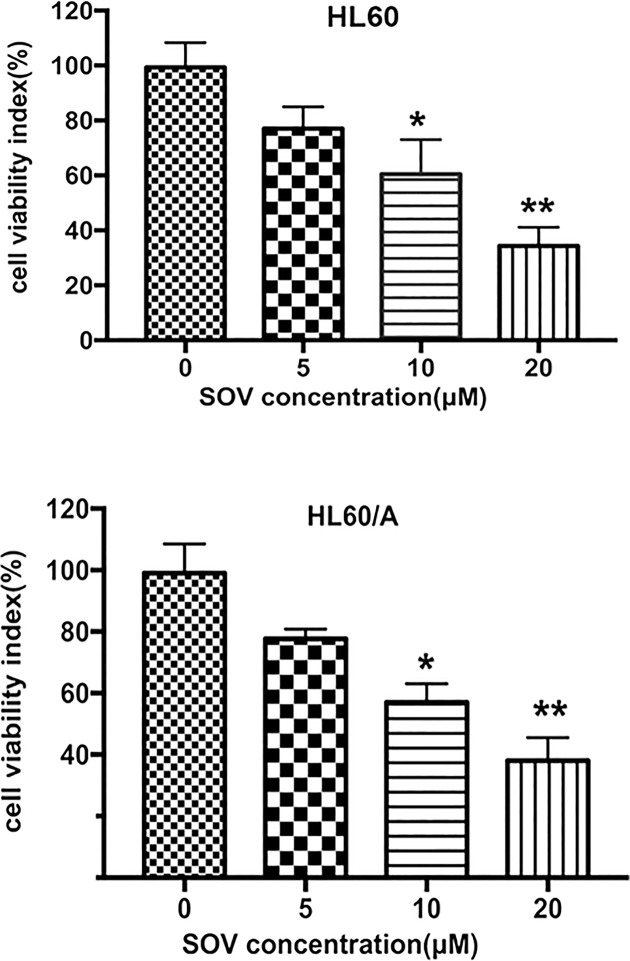
Cell growth *in vitro* As indicated, HL60 cells and HL60/A cells were incubated with SOV at various concentrations for 72 h. The cell viability index was determined by using a CCK-8 assay. * Indicates a significant difference at *P*<0.05, and ** a highly significant difference at *P*<0.01, compared with control.

HL60 cells and HL60/A cells were incubated with increasing concentrations of SOV (5, 10 and 20 μM) for 72 h, and cell viability was determined with a CCK-8 kit. Here we showed that SOV significantly suppressed the proliferation of HL60 cells and HL60/A cells in a dose-dependent manner (Figure 1). After 72 h treatment with SOV there was a significant difference in the cell viability index between control and 10 or 20 μM SOV-treated cells (*P*<0.05), whereas a lower dose of SOV (5 μM) also caused a slight but statistically not significant decrease in cell viability index, compared with control.

### SOV induces G_2_/M cell cycle arrest in HL60 cells and HL60/A cells

**Figure 2:**
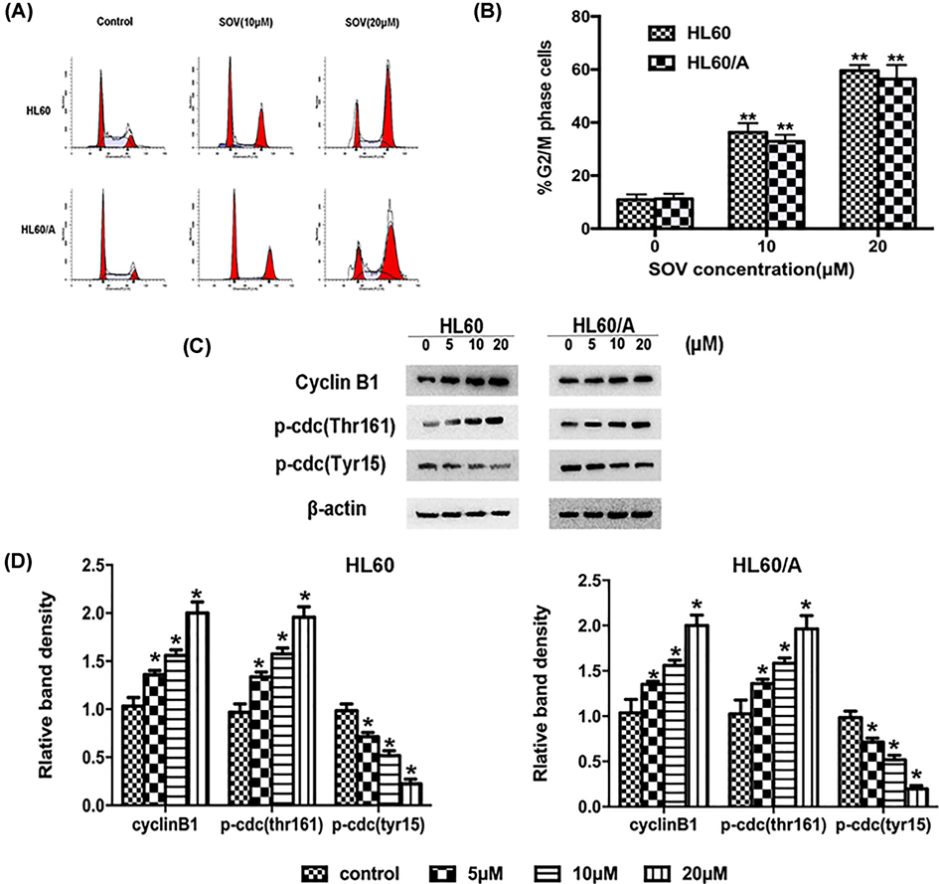
SOV induces G_2_/M cell cycle arrest in leukemia cells (**A**) DNA content and cell cycle analysis of SOV-treated cells. HL60 and HL60/A cells were incubated with 0, 10, 20 μmol/l SOV for 48 h. The cell cycle distribution was determined via flow cytometry. (**B**) Data from cell cycle distribution shown as a representative of at least three independent experiments and representative histograms are shown for cytometrically analyzed cells. A significant difference from SOV-treated cells is denoted by ‘**’ *P*<0.01. (**C**) Expression of G_2_/M cell cycle relative proteins Tyr^15^ and Thr^161^ phosphorylation of cdc2 and Cyclin B1 was determined via Western blot after treatment with SOV at various concentrations for 48 h. β-actin was used as internal control. (**D**) The density of each band from (**C**) was measured and compared with that of the internal control, β-actin. ‘*’ indicates significant difference (*P*<0.05) in band density between SOV-treated groups and control.

To explore the mechanisms of SOV-induced anti-cancer effects in HL60 cells and HL60/A cells, we first used a cell cycle kit to determine the percentage of cells in each cell cycle phase. Our results showed that SOV could cause cell cycle arrest at the G_2_/M phase in a dose-dependent manner in HL60 cells and HL60/A cells (Figure 2A,B). The Western blot results indicated that the expression of G_2_/M cell cycle regulating factors cyclin B1 and Thr^161^ phosphorylation of cdc2 showed a dose-dependent increase. On the other side, a decrease in Tyr^15^ phosphorylation of cdc2 was also observed in the same conditions (Figure 2C,D). These data suggest that the inhibition of cell proliferation by SOV is associated with the induction of G_2_/M phase arrest.

### SOV induces the apoptosis of HL60 cells and HL60/A cells

**Figure 3:**
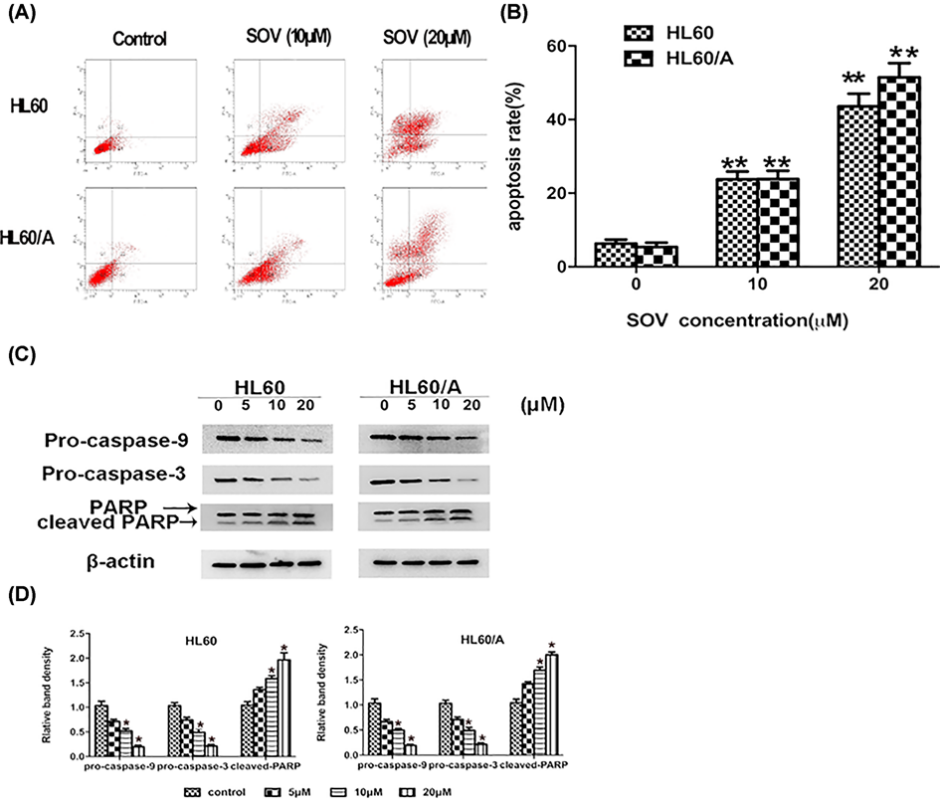
SOV induced cancer cell apoptosis (**A**) HL60 and HL60/A cells were treated with 0, 10 and 20 μM/l SOV for 48 h and harvested. Flow cytometry was performed to observe apoptosis rates. (**B**) Representative histograms from cytometrically analyzed the two cell lines treated with control and SOV. ‘**’ compared with control, *P*<0.01. (**C**) Western blot analysis on the expressions of pro-caspase-9, pro-caspase-3 and PARP from respective cell homogenates, with β-actin as the protein loading control. (**D**) The density of each Western blot protein band was measured and compared with that of the internal control, β-actin. ‘*’ indicates significant difference (*P*<0.05) in band density between SOV-treated groups and control.

HL60 cells and HL60/A cells were incubated with SOV at different concentrations for 48 h and then stained with Annexin V/PI, cell apoptosis was determined by flow cytometry. As shown in Figure 3A, HL60 cells and HL60/A cells have shown SOV-induced dose-dependent apoptosis, including early as well as late apoptotic cell death. The analysis demonstrated that >40% of the HL60 cells and HL60/A cells underwent apoptosis within 48 h after initiation of 20 μM SOV treatment. Then we further determined the levels of apoptosis-related proteins in these SOV-treated HL60 cells and HL60/A cells. As shown in Figure 3C,D, the SOV-treated HL60 cells and HL60/A cells exhibited a concentration-dependent down-regulation of pro-caspase-9 and 3, and an increase in cleaved PARP expression, which could be as another evidence of apoptosis induction. These results suggest that SOV induced the apoptosis of HL60 cells and HL60/A cells at least partly by activating caspases-3 and 9, and promoting PARP cleavage, therefore, the intrinsic mitochondrial apoptosis pathway might be involved in SOV-induced apoptosis.

### SOV diminishes ΔΨm of HL60 cells and HL60/A cells

**Figure 4:**
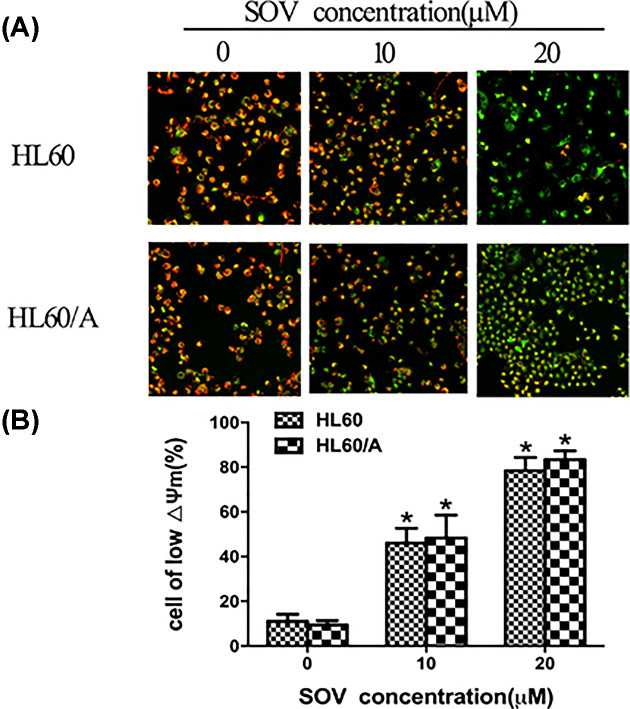
Changes in mitochondrial membrane potential *in vitro* (**A**) Cells subjected to SOV for different doses (0, 10 and 20 μM) were stained by JC-1. Change of ΔΨm was detected by fluorescence microscopy. Healthy cells that have high ΔΨm show punctuate yellow fluorescence. Apoptosis cells show diffuse green fluorescence because of a decrease in ΔΨm. Bar = 100 μm. (**B**) Representative histograms are shown for cytometrically analyzed cells labeled with the JC-1 dye. *P<0.01, statistical significance in SOV treated groups compared with the control.

Disruption of ΔΨm is one of the earliest intracellular events that occur following the induction of apoptosis [22]. To confirm the involvement of mitochondria during SOV-induced apoptosis, we investigated the changes in ΔΨm of HL60 cells and HL60/A cells after a 48-h incubation with 10 and 20 μM SOV. As shown in Figure 4A, SOV significantly (*P*<0.01) diminished the ΔΨm compared with control in two cell lines, representative histograms for the above cells are shown in Figure 4B, the analysis demonstrated that the ΔΨm reduced was more apparent along with increasing concentrations.

### SOV suppresses autophagy in HL60 cells and HL60/A cells

**Figure 5:**
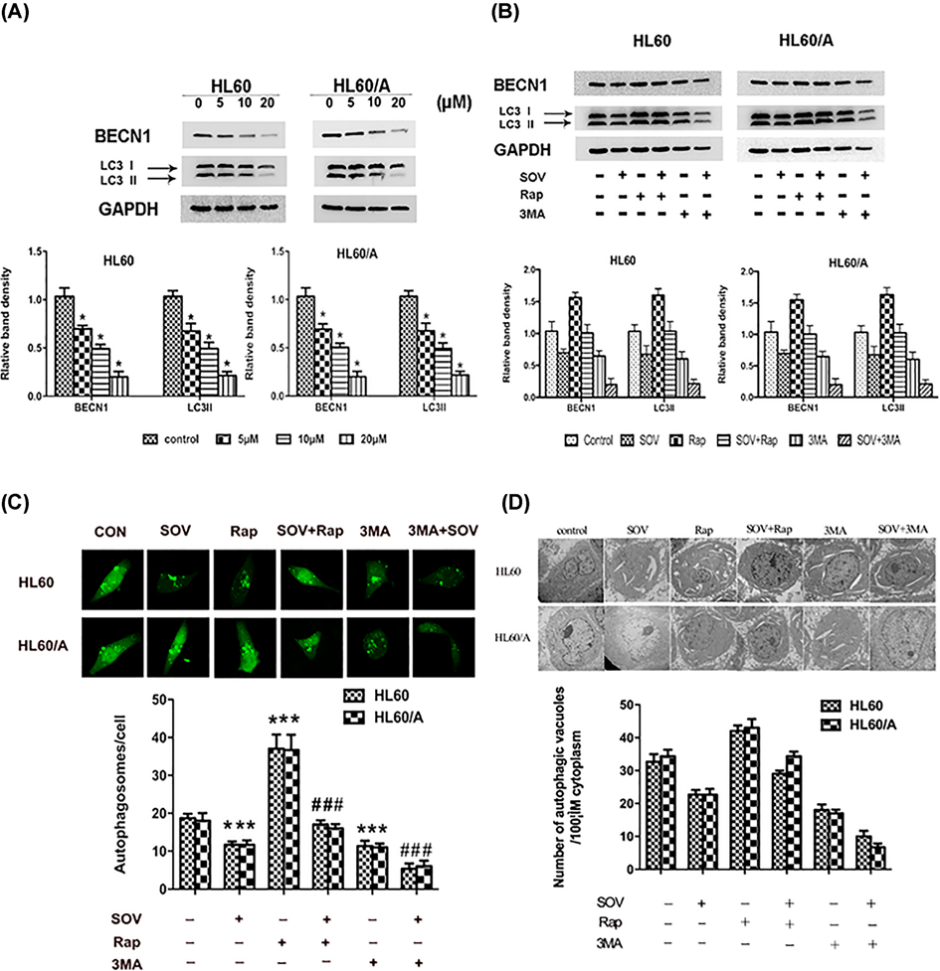
The activity of autophagy during the treatment of SOV in leukemia cells *in vitro* (**A**) Expression of autophagy relative proteins BECN1 and LC3-II were determined via Western blot after treatment with SOV at various concentrations for 48 h. GAPDH was used as internal control. The density of each Western blot protein band was measured and compared with GAPDH. (**B**) Western blots analysis of the expression of LC3-II and BECN1 in leukemia cells that were subjected to control, SOV, Rap, Rap+SOV, 3MA, 3MA+SOV at 48 h after treatment, with GAPDH as the protein loading control. The density of each band was measured and compared with GAPDH. (**C**) The average number of autophagosomes/cell ± SD counted from confocal microscopy images of leukemia cells expressing GFP-LC3 in (B). Bar = 10 μm. (**D**) Representative electron micrographs showing autophagic vacuoles in leukemia cells in (B) and the quantification of the number of autophagic vacuoles per 100 μm cytoplasm. Data are expressed as mean ± SD. Bar = 2 μm. *Significant difference from control, *P*<0.05; *** and ^###^significant difference from SOV group, *P*<0.05. Abbreviation: Rap, rapamycin.

To further investigate the anti-tumor mechanisms of SOV, we examined the effect of SOV on autophagy in HL60 cells and HL60/A cells. The expression of LC3 and BECN1 in HL60 cells and HL60/A cells had been detected by Western blotting. The results showed a dose-dependent decrease in the levels of LC3-II and BECN1 in comparison with control (Figure 5A,B), indicating the inhibition of autophagy caused by SOV. We further detected autophagy by analyzing the formation of fluorescent puncta or autophagosomes in GFP–LC3-transfected HL60 cells and HL60/A cells. Some autophagosomes were detected, as characterized by punctate, green-fluorescing structures. As shown in Figure 5C, most control HL60 cells and HL60/A cells had an even and diffused GFP–LC3 staining with occasional puncta, whereas SOV markedly decreased the number of autophagosomes in HL60 cells and HL60/A cells. We also detected the autophagosomes and related autophagic vacuoles by electron microscopy (Figure 5D), the typical autophagosomes being characterized by double-or-multiple-membrane structures containing cytoplasm or undigested organelles such as mitochondria, while the autolysosomes were identified as single-membrane structures with remnants of cytoplasmic components. The autophagic vacuoles were evaluated by morphometric methods. The amount of autophagic vacuoles per unit cytoplasmic area of 100 mm^2^ was evaluated. Compared with the control, fewer autophagic vacuoles were seen in SOV-treated HL60 cells and HL60/A cells. Thus, autophagy is suppressed by SOV.

### SOV-induced autophagy inhibition effect plays a prodeath role in HL60 cells and HL60/A cells *in vitro*

**Figure 6:**
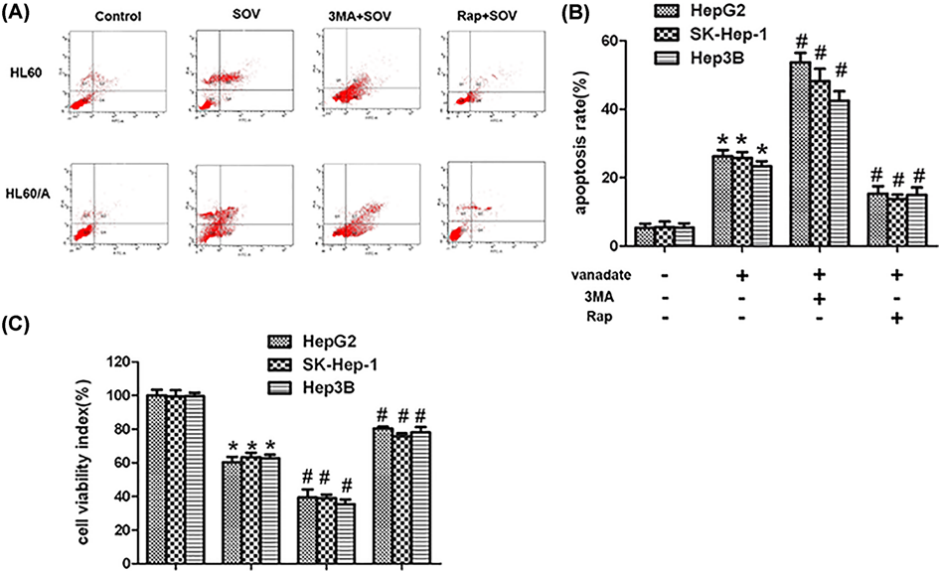
SOV inhibits autophagy and promotes apoptosis (**A**) Cell viabilities of leukemia cells that were treated with control, SOV, Rap+SOV, 3MA+SOV were determined at 72 h after treatment. Data are expressed as mean ± SD. (**B**) HL60 and HL60/A cells were treated with control, SOV, Rap+SOV, 3MA+SOV for 48 h. Flow cytometry was performed to observe apoptosis rates. (**C**) Representative histograms from cytometrically analyzed the two cell lines treated with control, SOV, Rap+SOV, and 3MA+SOV. *Significant difference from control, *P*<0.05; ^#^significant difference from SOV group, *P*<0.05.

To further evaluate the role of autophagy in SOV-treated cells, rapamycin and 3MA were used to reverse and enhance the SOV-induced autophagy inhibition, respectively. Their autophagy regulatory roles in HL60 cells and HL60/A cells were confirmed by Western blot (Figure 5B) and autophagosomes detection (Figure 5C,D). As shown in Figure 6, 3MA significantly enhanced proliferation inhibition and the apoptosis induction caused by SOV, while rapamycin did the opposite effect (Figure 6A–C). These results indicated that SOV-induced autophagy inhibition effect plays a prodeath role in HL60 cells and HL60/A cells *in vitro*.

## Discussion

The present study has demonstrated the anti-cancer effects of SOV in the treatment of human HL60 cells and HL60/A cells. SOV inhibited the growth of HL60 cells and HL60/A cells in a dose-dependent manner. The underlying mechanisms may be related to regulations of proliferation, cell cycle and apoptosis. Upon further exploration, we found that for the first time SOV might act as a novel autophagy inhibitor in cancer therapy.

In recent years, significant progress has been made in the anti-leukemia mechanisms of drugs. Among them, inhibition of proliferation, inducing apoptosis and autophagy are the main anti-tumor mechanisms. Vanadium salts have high biological significance as an antineoplastic drug. There have been many reports on the anti-tumor effect, such as lung, kidney and prostate cancer [23,24]. Here we demonstrated that SOV inhibited the growth of HL60 cells and HL60/A cells. Woo et al. showed that SOV caused G_2_/M phase cell cycle arrest in Chinese hamster ovary cells [25]. Our results show that SOV could induce G_2_/M phase cell cycle arrest in HL60 cells and HL60/A cells line.

In addition, the observation of the cell cycle related protein levels showed that, after SOV treatment, the cyclin B1 and phosphorylated Thr^161^ increased with increasing dose, while the phosphorylated Tyr^15^ decreased with dose, both of which were previously a prerequisite for the activation of cdc2 kinase at the G_2_/M phase. Since the cyclin B1/cdc2 kinase plays a vital role as M-phase promoting factor in the G_2_/M transition, our results suggested that the SOV anti-tumor mechanisms have a close relationship with G_2_/M arrest.

In recent years, studies on the mechanism of apoptosis occurrence and regulation show that the three major apoptotic pathways are the mitochondrial and death receptor pathways, endoplasmic reticulum signal transduction pathway [26]. The released cytochrome *c* from mitochondria to the cytosol binds to Apaf-1, resulting in proteolytic processing and activation of caspase-9. Subsequently, activated caspase-9 then activates caspase-3, triggering a cascade of additional caspase activation that culminates in apoptosis [27].

Although most anti-tumor drugs can induce apoptosis in cancer cells, the mechanism is not yet precise. Previous studies have a disagreement about the relationship between vanadate and apoptosis [28–31].

Our research showed that SOV-induced apoptosis in HL60 cells and HL60/A cells. In the present study, we demonstrated that the activation of caspases and PARP were involved in the SOV-induced apoptosis. We have also shown that SOV reduces the cell line plastochondria membrane potential, which leads to enhanced activation of caspase-9 and -3. Therefore, the effects of SOV in inducing the apoptosis of HL60 cells and HL60/A cells may involve the mitochondrial pathway.

Autophagy is an evolutionarily conserved process that involves lysosomal degradation of cytoplasmic and cellular [32,33], which occurs in all eukaryotic cells from yeast to mammals [34–36]. This process is believed to be important in the progression of cancers. However, the link between autophagy and cancer is often considered controversial. Liu et al. studies have showed that induction of autophagy could promote tumor cell death [37] while Longo et al. have demonstrated that autophagy inhibition can potentiate the anti-tumor effect in hepatocellular carcinoma [38]. Here our results showed that SOV can inhibit autophagy, which might enhance the effect of chemotherapeutic drugs in subsequent studies. Further reduction in autophagy by 3MA can significantly enhance the apoptosis of HL60 cells and HL60/A cells induced by SOV, while rapamycin can reverse such autophagy inhibition and reduce the apoptosis-inducing effect of SOV in HL60 cells and HL60/A cells, these data indicate that such autophagy inhibition effect plays a prodeath role.

In summary, for the first time, we found that SOV has significant anti-cancer effects against human HL60 cells and HL60/A cells. Our results suggest that the underlying mechanisms may be, at least in part, due to SOV inhibits the proliferation and induces the mitochondria-dependent apoptosis and G_2_/M cell cycle arrest of HL60 cells and HL60/A cells. Through further studies, we found that SOV could also inhibit autophagy in HL60 cells and HL60/A cells, which may play a prodeath role. The demonstrated activities of SOV support its further evaluation as a treatment for human leukemia.

## Abbreviations

3MA: 3-methyladenine
AML: acute myeloid leukemia
APL: acute promyelocytic leukemia
ATO: arsenic trioxide
ATRA: all-trans retinoic acid
CCK-8: Cell Counting Kit-8
GAPDH: glyceraldehyde-3-phosphate dehydrogenase
LC3: microtubule-associated protein 1A/1B-light chain 3
MMP: mitochondrial membrane potential
PARP: poly (ADP-ribose) polymerase
Rap: rapamycin
SOV: sodium orthovanadate
